# Age-related differences in staging, treatment and net survival in relation to frailty in adults with colon cancer in England: an analysis of the COloRECTal cancer data repository (CORECT-R) resource

**DOI:** 10.1093/ageing/afaf025

**Published:** 2025-02-19

**Authors:** Sophie Pilleron, Rebecca J Birch, John Taylor, Shane O'Hanlon, Eva J A Morris

**Affiliations:** Nuffield Department of Population Health, University of Oxford, Old Road Campus, Oxford OX1 2JD, UK; Department of Precision Health, Luxembourg Institute of Health – Ageing, Cancer, and Disparities Research Unit, Department of Precision Health, Luxembourg Institute of Health, 1A-B, rue Thomas Edison, Strassen, L1445, Luxembourg; Leeds Institute of Medical Research at St James's, University of Leeds, Leeds, UK; University of Leeds, Leeds LS2 9JT, UK; Mater Private Hospital, 64 Eccles St, Dublin D07 Y5N7, Ireland; School of Medicine, University College Dublin, Dublin D04 V1W8, Ireland; Nuffield Department of Population Health, University of Oxford, Old Road Campus, Oxford OX1 2JD, UK

**Keywords:** frailty, neoplasm, epidemiology, population-based, colon, older people

## Abstract

**Objective:**

To describe the distribution of disease stages, receipt of major surgery, 1-year net survival (NS) and 1-year conditional NS in relation to age and frailty in adults aged ≥50 diagnosed with colon cancer in England.

**Methods:**

We obtained data on adults aged 50–99 diagnosed with colon cancer between 2014 and 2019, followed up through December 2021, from the national population-based COloRECTal cancer Repository. Frailty was assessed using the Secondary Care Administrative Records Frailty (SCARF) index categorised into fit, mild, moderate and severe frailty. Data on major resection were obtained through linkage with Hospital Episode Statistics dataset. Major resection rates were calculated in adults with stage I–III cancer. Descriptive statistics were used as appropriate. One-year NS from cancer diagnosis and 1-year conditional NS were estimated using the Pohar-Perme estimator.

**Results:**

Out of 130 360 individuals (48% females—50% over 75), 48.9% were fit, ranging from 69% in the 50–64 age group to 31% in the 85–99 age group. Over 80% of adults with stage I–III cancer underwent a major resection. This percentage was 58% amongst fit adults aged over 85. One-year NS decreased as age increased across all frailty levels. Differences in NS between the 50–64 age group and the 85–99 age group were reduced in adults who survived beyond 1 year from diagnosis except for severely frail adults.

**Conclusion:**

This population-based study shows that a non-negligible proportion of older adults diagnosed with colon cancer and deemed ‘fit’ through the SCARF did not receive surgery that may impact their survival.

## Key Points

- Most population-based studies uses chronological age when describing the epidemiology of cancer in older populations.
- Frailty can be assessed in electronic health data using recently developed indices such as Secondary Care Administrative Records Frailty (SCARF) index.
- Under half of adults aged 50–99 diagnosed with colon cancer in England over 2014–2019 were considered fit.
- Whilst otherwise fit, 58% of those aged 85–99 with stage I-III colon cancer did not undergo major surgery.
- We confirmed poorer cancer survival in older adults, after correcting for background mortality, mainly within the first year.

## Introduction

Colon cancer is common with over 24 000 new diagnoses every year in England, and survival is relatively good with 76% surviving the first year of diagnosis [1]. However, a study conducted in high-income countries, including England, showed adults aged 75 years or older had poorer colon cancer survival than younger adults, even after removing age-related differences in background mortality [2]. Previous analyses highlighted an excess mortality in the first months of diagnosis in older adults that was greater in England when compared to Norway, Finland and Sweden [3, 4]. Together with studies in other settings [4–6], these findings suggest the period around diagnosis and first-line treatment is crucial in understanding age-related differences in colon cancer survival.

Surgery is the main treatment in adults with non-metastatic colon cancer regardless of their age [7]. However, the likelihood of receipt of surgery decreases as age at diagnosis increases, paralleling the poorer colon cancer survival, especially in adults aged 75 years or older [8]. Reasons for not performing surgery include patients’ preferences [9, 10], clinical condition (e.g. the patient is too frail, or at high risk of postoperative complications) or age bias [11, 12].

Older adults are more likely to be diagnosed at an advanced stage or through emergency settings than younger adults, and both are associated with a lower chance of curative treatment and an excess mortality risk [13, 14].

All these population-based studies used chronological age only; yet, the same cohort of older adults can include people with health status ranging from very poor to very good [15]. If we are to further understand the reasons for age-related differences in survival outcomes at population level, there is a need to have an indicator of biological/physiological age that reflects this range in health status.

From electronic health data, the level of frailty may be assessed using recently developed indices, including the Secondary Care Administrative Records Frailty (SCARF) index [16] that is based on the cumulative deficit model of frailty [17].

Investigating age-related differences in cancer treatment and outcomes by frailty level may help quantify the extent to which the differences are due to inequity of management.

Using the national population-based COloRECTal cancer Repository (CORECT-R), this study describes differences in: distribution of disease stage at diagnosis, receipt of major surgery, 1-year net survival and 1-year net survival conditional on surviving the first year of diagnosis, in relation to age and frailty status in adults aged 50 years or older diagnosed with colon cancer in England.

## Methods

This retrospective observational population-based study included 130 377 adults diagnosed in England with a first occurrence of colon cancer (International Classification of Disease-version 10 (ICD10) codes: C18.0-C18.9) between 2014 and 2019 aged between 50 and 99 years old [18]. These data were sourced from CORECT-R, a national population-based resource that provides information about all people diagnosed with colorectal cancer in England by linking cancer registry data to a variety of datasets including hospitalisation data from Hospital Episode Statistics (HES) [18]. Analysis was restricted to individuals aged ≥50 because early onset colon cancer has different features and management [19, 20].

We excluded adults with no information on vital status (n = 2) or the last known alive date (n = 115).

Patient characteristics [age at diagnosis, sex, socio-economic deprivation level measured using quintiles of the income domain of the 2015 Index of Multiple Deprivation (IMD)] [21], stage at diagnosis (categorised into I, II, III, IV and unknown), vital status (updated in July 2022) and date of death were retrieved from the National Cancer Registration and Analysis Service (NCRAS) dataset component of CORECT-R.

The SCARF index was calculated from the linked HES data from 32 deficits covering elements of the comprehensive geriatric assessment (excluding polypharmacy) and 20 comorbidities (see list of items in Supplemental Material) [16]. It was validated in women aged ≥50 years old diagnosed with breast cancer in 2014–17 in England. The index was built for each patient using the number of deficits (out of 32) identified within 2 years prior to cancer diagnosis. A recent study showed that SCARF performs as well as other frailty indices using HES data in English colorectal cancer patients [22].

### Statistical analysis

Based on the original study, the SCARF index was stratified into four levels of frailty as follows: fit = 0–1 deficit, mild frailty = 2–3 deficits, moderate frailty = 4–5 deficits and severe frailty = 6 or more deficits [16]. We categorised age at diagnosis into the four following categories: 50–64; 65–74; 75–84; 85–99.

All analyses were stratified by age group and SCARF category. Percentages were used to describe categorical variables and median and interquartile range for continuous variables. We calculated major resection rates in adults with stage I–III colon cancer only as surgery can be complicated in advanced metastatic cancer [23]. One-year net survival, that is survival in the hypothetical world where cancer is the only cause of death, and 1-year conditional survival was estimated using the Pohar-Perme estimator from date of diagnosis and date of diagnosis +365 days, respectively [24, 25]. The Pohar Perme estimator is based on the calculation of the excess mortality hazard attributable to cancer and requires two key pieces of information: the mortality hazard for cancer patients, sourced from the CORECT-R resource, and the background mortality rates in the general population. The background mortality, i.e. the probability that a person of a certain sex, and age at the beginning of year *y* survived until the end of that year, was obtained from population lifetables. Sub-national smoothed lifetables stratified by age, sex, socio-economic deprivation level and region for the period 2002–2021 were retrieved from https://www.cancerdata.nhs.uk/survival/lifetables. The national probabilities were estimated by calculating the weighted average of regional probabilities by deprivation level, sex, age and year. The weights were the relative region’s population size obtained from Office for National Statistics by year (up to 2020), age (up to 90+), sex and deprivation level. We assumed the weight for ages 91–100 was the weight for the age category 90+, and the weights for the year 2021 were similar to those of the year 2020. We censored survival time at 1 year after the start of follow-up (i.e. diagnosis date or diagnosis date +1 year for the estimation of net survival and conditional net survival, respectively). The end of follow-up was set on 31 December 2021 due to availability of life tables required for the analysis.

We performed data management and statistical analyses using R statistical software (version 2022.07.2, Build 576; R Development Core Team, 2022). The relsurv package was used for estimating the Pohar-Perme estimator [25].

### Ethics

The project is covered by the Establishing a UK Colorectal Cancer Intelligence Hub Research Ethics approval (18/SW/1034) granted by the Southwest—Central Bristol Research Ethics Committee on the 1 June 2018.

## Results

The study population included 130 260 adults aged ≥50 diagnosed with colon cancer in 2014–2019 in England (48.0% female—50.2% over 75).

Overall, 13.5% were diagnosed with stage I, 26.2% with stage II, 24.5% with stage III, 23.9% with stage IV and 12.0% had an unknown stage. The percentage of cancers with unknown stage increased as age increased from 5.9% in the 50–64 age group to 28.6% in the 85–99 age group. In contrast, the percentages of adults diagnosed with stage I cancer decreased as age increased from 15.3% to 7.9% between the youngest and the oldest age group (Table 1). The percentages of the other stages were similar across age groups.

**Table 1:** Characteristics of adults aged ≥50 diagnosed with colon cancer in 2014–18, England.

**Age groups**	**50–64**	**65–74**	**75–84**	**85–99**
n	26 686	38 122	42 797	22 655
# deaths within 1st year (%)	4454 (16.7)	8310 (21.8)	14 541 (34.0)	12 846 (56.7)
Median age (Interquantile Range)	59 (55–62)	70 (68–72)	80 (77–82)	88 (86–90)
Females (%)	11 995 (44.9)	16 722 (43.9)	20 843 (48.7)	12 966 (57.2)
Deprivation quintile (%)				
1—most deprived	5654 (21.2)	8566 (22.5)	9820 (22.9)	5171 (22.8)
2	5852 (21.9)	9005 (23.6)	10 064 (23.5)	5297 (23.4)
3	5307 (19.9)	7819 (20.5)	9014 (21.1)	5008 (22.1)
4	4978 (18.7)	6861 (18.0)	7598 (17.8)	4145 (18.3)
5—least deprived	4895 (18.3)	5871 (15.4)	6301 (14.7)	3034 (13.4)
Stage at diagnosis (%)				
I	4088 (15.3)	6365 (16.7)	5312 (12.4)	1786 (7.9)
II	6529 (24.5)	10 431 (27.4)	12 219 (28.6)	4904 (21.6)
III	7385 (27.7)	9857 (25.9)	10 351 (24.2)	4364 (19.3)
IV	7102 (26.6)	8909 (23.4)	9936 (23.2)	5132 (22.7)
Unknown	1582 (5.9)	2560 (6.7)	4979 (11.6)	6469 (28.6)
Frailty level (%)				
Fit	18 406 (69.0)	21 621 (56.7)	16 770 (39.2)	6964 (30.7)
Mildly frail	5112 (19.2)	8386 (22.0)	10 011 (23.4)	4355 (19.2)
Moderately frail	2087 (7.8)	4451 (11.7)	7401 (17.3)	4118 (18.2)
Severely frail	1081 (4.1)	3664 (9.6)	8615 (20.1)	7218 (31.9)
Major resection^a^ (%)	15 823 (87.9)	23 385 (87.8)	22 690 (81.4)	6220 (56.3)
1-year net survival (%–95% confidence interval)	84.4 (83.9–84.8)	81.0 (80.5–81.4)	71.8 (71.4–72.3)	53.5 (52.7–54.3)

^a^Restricted to adults diagnosed with stage I–III colon cancer.

**Figure 1:**
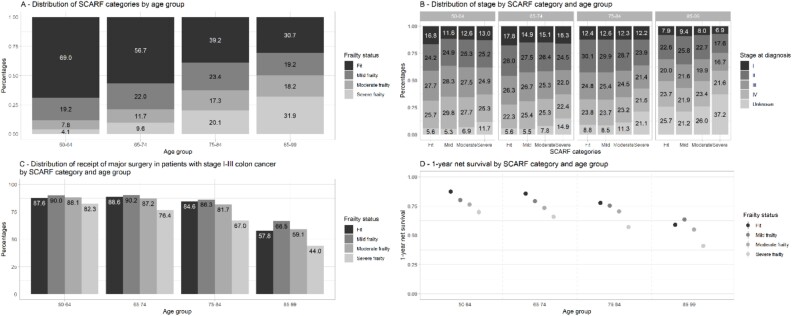
Distribution of (A) SCARF categories by age group, (B) stage at diagnosis, (C) receipt of major surgery in adults with stage I–III cancer and (D) 1-year net survival by SCARF category and age group in adults aged ≥50 diagnosed with colon cancer in 2014–19, England.

Based on the SCARF index, 48.9% of adults were categorised as fit, 21.4% as mildly frail, 13.9% as moderately frail and 15.8% were severely frail. The percentage of fit adults decreased as age increased from 69% in the 50–64 age group to 31% in the 85–99 age group (Table 1 and Figure 1A), whilst the percentage of severely frail adults increased from 4% in the 50–64 age group to 32% in the oldest group.

Overall, 81.5% of adults with stage I–III colon cancer underwent a major resection. This percentage was the smallest in adults aged 85–99 with 56.3% who underwent major resection against >80% in younger age groups (Table 1).

One-year net survival from colon cancer decreased as age increased from 84.4% (83.9%–84.8%) in the 50–64 age group to 53.5% (52.7%–54.3%) in the oldest age group (Table 1).

Across all age groups, the percentage of people diagnosed with colon cancer of unknown stage was greatest in those with severe frailty whilst they were similar in all other frailty levels (Figure 1B). The percentage of adults with unknown stage was particularly high in those aged 85–99, ranging from 26% in fit adults to 37% in severely frail adults.

Major resection rates were lowest in adults aged ≥85 in all frailty levels (Figure 1C). Age-related differences in major resection rates between the 50–64-year-old group and the 85–99-year-old group varied between 23.5 percentage-points in mildly frail adults and 38.3 percentage-points in those with severe frailty. Differences in other frailty levels were 29.8 and 29.0 percentage-points in fit and moderately frail adults, respectively.

With respect to survival, the 1-year net survival decreased as age increased across all frailty levels (Figure 1C, Supplemental Table 1). Except for adults with severe frailty, age-related differences in 1-year net survival were greatly reduced across all frailty levels when conditional on surviving the first year of diagnosis (Supplemental Table 1 and Figure 1). In those who survived the first year, oldest adults still had poorer colon cancer survival than younger age groups.

## Discussion

Using national population-based cancer registry data in England, we showed that just under half of adults aged 50–99 diagnosed with colon cancer over the study period were considered fit according to the SCARF classification. However, whilst otherwise fit, 58% of those aged 85–99 with stage I-III colon cancer did not undergo major surgery. We also confirmed poorer cancer survival in older adults than younger adults, after mortality background differences were removed, mainly within the first year of diagnosis. Our findings call for a better understanding of the reasons for age-related differences in major surgical intervention rates in fit older adults with colon cancer in England.

Half of adults aged ≥50 diagnosed with colon cancer live with some degrees of frailty, that is much higher than the prevalence observed in the ELSA wave eight study (non-cancer) population of the same age (9.9% pre-frailty and 8.1% frailty—different frailty measure) [26] or in women aged ≥50 diagnosed with breast cancer (20.8%—SCARF index) [16]. Although comparison between different populations is difficult, the higher prevalence of frailty in adults with colon cancer was unexpected. One hypothesis may be that both conditions share similar risk factors, specifically lifestyle risk factors (i.e. high body mass index) [27]. Another hypothesis is that the development of colon cancer over time increases the vulnerability of the body to face with stressors. Further work is needed to investigate reasons for higher prevalence of frailty in older adults with colon cancer.

As in previous studies [28], we confirmed that the percentages of unknown stage at diagnosis increased as age increased in adults diagnosed with colon cancer in England. However, we showed, for the first time, that unknown stage was more frequent in frailer adults regardless of age at diagnosis. We also showed that fit older adults diagnosed with colon cancer of unknown stage were less likely to receive surgery than their younger counterparts. However, we are unable to know reasons for unstaged cancer, specifically in older ones, with data available. They could be because adults were seemed unfit to undergo surgery at the time of diagnosis, or surgery was not proposed because of potential age bias, or the patient refused it.

Whilst it is well documented that older age is associated with lower receipt of surgery [8], our population-based study showed that even fit older adults (measured through the SCARF index) were less likely to receive surgery compared to fit younger adults. Not receiving surgery may be a patient choice or physicians’ decision based on patients’ health status or because of a perceived lack of evidence base and specific recommendations for older adults. Surgery may offer a survival benefit for fit older adults with colon cancer, therefore there is an urgent need to explore why this group do not receive surgery.

More advanced disease and lower major surgery receipt in older adults logically resulted in poorer 1 year colon cancer survival in these adults compared to younger adults. Age-related differences in colon cancer survival have already been documented in high-income countries [3, 29, 30]; however, our findings showed that these differences exist even in seemingly fit adults.

Our study has limitations. There may be some misclassifications into frailty levels using SCARF index. Indeed, because SCARF index is built using hospital discharge data, it is possible that adults with some degrees of frailty who did not present to hospital in the previous 2 years were incorrectly categorised into fit adults. In addition, some deficits may not have been recorded. On the contrary, SCARF items are considered regardless of their duration (e.g. transitory or chronic). Triangulation with other electronic data sources such as general practitioner data or pharmaceutical prescription data is warranted. An in-depth understanding of factors influencing age-related differences in the treatment-decision making process using qualitative approach could also be valuable, but this type of research is still scarce.

Another limitation relies upon the use of the same weight for every SCARF index item that may not have the same impact on colon cancer management or survival.

A further limitation is that the lifetable used to estimate net survival was not stratified by frailty status that is associated to both colon cancer specific- and all-cause mortality. Net survival estimates may then be under—or over-estimated.

Beyond survival, we have not examined other outcomes such as postoperative morbidity and quality of life. Future studies are needed to explore these outcomes in relation to frailty status.

The SCARF index, like other frailty indices developed using administrative datasets, is not intended for use in clinical practice. Consequently, its performance in comparison to clinically used tools, such as the WHO performance status or the Clinical Frailty Scale, remains unclear. Future studies should focus on comparing tools developed from electronic healthcare data with those used in practice to determine if they are assessing the same aspects of frailty.

As strengths, our study is nationally inclusive, reducing the risk for selection bias. The incorporation of a frailty measure also provides more clinically relevant analysis.

## Conclusion

In this nationally inclusive population-based study, we showed that a non-negligible proportion of fit older adults diagnosed with colon cancer did not get their cancer staged, and did not receive major resection, both factors associated with poorer colon cancer survival. A comprehensive understanding of drivers of age-related differences in staging and treatment in fit adults is urgently needed.

## Supplementary Material

aa-24-0997-File003_afaf025

## Data Availability

CORECT-R data are available upon request at https://www.ndph.ox.ac.uk/corectr/corect-r.

## References

[1] Cancer Data . [cited 2022 Dec 13]. Available from: https://digital.nhs.uk/data-and-information/publications/statistical/cancer-registration-statistics.

[2] Araghi M, Arnold M, Rutherford MJ, Guren MG, Cabasag CJ, Bardot A et al. Colon and rectal cancer survival in seven high-income countries 2010–2014: Variation by age and stage at diagnosis (the ICBP SURVMARK-2 project). Gut 2021;70:114–26. 10.1136/gutjnl-2020-320625 32482683

[3] Pilleron S, Charvat H, Araghi M et al. Age disparities in stage-specific colon cancer survival across seven countries: An international cancer benchmarking partnership SURVMARK-2 population-based study. Int J Cancer. 2021;148:1575–85. 33006395 10.1002/ijc.33326

[4] Morris EJA, Sandin F, Lambert PC et al. A population-based comparison of the survival of patients with colorectal cancer in England, Norway and Sweden between 1996 and 2004. Gut. 2011;60:1087–93. 21303917 10.1136/gut.2010.229575

[5] Withrow DR, Nicholson BD, Morris EJA et al. Age-related differences in cancer relative survival in the United States: A SEER-18 analysis. Int J Cancer. 2023;152:2283–91. 10.1002/ijc.34463 36752633

[6] Colonna M, Bossard N, Remontet L et al. Changes in the risk of death from cancer up to five years after diagnosis in elderly patients: A study of five common cancers. Int J Cancer. 2010;127:924–31. 19998335 10.1002/ijc.25101

[7] Papamichael D, Audisio RA, Glimelius B et al. Treatment of colorectal cancer in older patients: International Society of Geriatric Oncology (SIOG) consensus recommendations 2013. Ann Oncol. 2015;26:463–76. 25015334 10.1093/annonc/mdu253

[8] Majano SB, Girolamo CD, Rachet B et al. Surgical treatment and survival from colorectal cancer in Denmark, England, Norway, and Sweden: A population-based study. Lancet Oncol. 2019;20:74–87. 30545752 10.1016/S1470-2045(18)30646-6 PMC6318222

[9] Dias LM, Bezerra MR, Barra WF et al. Refusal of medical treatment by older adults with cancer: A systematic review. Ann Palliative Med. 2021;10:4868877–4877. 10.21037/apm-20-2439 33832317

[10] Puts MTE, Tapscott B, Fitch M et al. A systematic review of factors influencing older adults’ decision to accept or decline cancer treatment. Cancer Treat Rev. 2015;41:197–215. 25579752 10.1016/j.ctrv.2014.12.010

[11] Neal D, Morgan JL, Kenny R, Ormerod T, Reed MWR. Is there evidence of age bias in breast cancer health care professionals’ treatment of older patients? Eur J Surg Oncol 2022;48:2401–7. 10.1016/j.ejso.2022.07.003 35871030

[12] Haase KR, Sattar S, Pilleron S et al. A scoping review of ageism towards older adults in cancer care. J Geriatr Oncol. 2023;14:101385. 10.1016/j.jgo.2022.09.014 36244925

[13] Barclay ME, Abel GA, Greenberg DC et al. Socio-demographic variation in stage at diagnosis of breast, bladder, colon, endometrial, lung, melanoma, prostate, rectal, renal and ovarian cancer in England and its population impact. Br J Cancer. 2021;124:1320–9. 33564123 10.1038/s41416-021-01279-z PMC8007585

[14] Zhou Y, Abel GA, Hamilton W et al. Diagnosis of cancer as an emergency: A critical review of current evidence. Nat Rev Clin Oncol. 2017;14:45–56. 27725680 10.1038/nrclinonc.2016.155

[15] Wildiers H, Heeren P, Puts M et al. International Society of Geriatric Oncology consensus on geriatric assessment in older patients with cancer. J Clin Oncol. 2014;32:2595–603. 25071125 10.1200/JCO.2013.54.8347 PMC4876338

[16] Jauhari Y, Gannon MR, Dodwell D et al. Construction of the secondary care administrative records frailty (SCARF) index and validation on older women with operable invasive breast cancer in England and Wales: A cohort study. BMJ Open. 2020;10:e035395. 10.1136/bmjopen-2019-035395 PMC7223146 32376755

[17] Rockwood K, Mitnitski A. Frailty in relation to the accumulation of deficits. J Gerontol A Biol Sci Med Sci. 2007;62:722–7. 17634318 10.1093/gerona/62.7.722

[18] Downing A, Hall P, Birch R et al. Data resource profile: The COloRECTal cancer data repository (CORECT-R). Int J Epidemiol. 2021;50:1418–1418k. 34255059 10.1093/ije/dyab122 PMC8580263

[19] Willauer AN, Liu Y, Pereira AAL et al. Clinical and molecular characterization of early-onset colorectal cancer. Cancer. 2019;125:2002–10. 10.1002/cncr.31994 PMC6583775 30854646

[20] Mauri G, Sartore-Bianchi A, Russo AG et al. Early-onset colorectal cancer in young individuals. Mol Oncol. 2019;13: 109–31. 30520562 10.1002/1878-0261.12417 PMC6360363

[21] Smith T, Noble M, Noble S, Wright G, McLennan D, Plunkett E. The English indices of deprivation 2015 - technical report. London, United Kingdom: Department for Communities and Local Government; 2015 [cited 2022 Jun 30] p. 126. Available from: https://assets.publishing.service.gov.uk/government/uploads/system/uploads/attachment_data/file/464485/English_Indices_of_Deprivation_2015_-_Technical-Report.pdf

[22] Birch R, Taylor J, Rahman T et al. A comparison of frailty measures in population-based data for patients with colorectal cancer. Age and Ageing. 2024;53:afae105. 10.1093/ageing/afae105 PMC11116828 38783754

[23] Cervantes A, Adam R, Roselló S, Arnold D, Normanno N, Taïeb J., et al. Metastatic colorectal cancer: ESMO clinical practice guideline for diagnosis, treatment and follow-up. Ann Oncol 2023;34:10–32. 10.1016/j.annonc.2022.10.003 36307056

[24] Roche L, Danieli C, Belot A et al. Cancer net survival on registry data: Use of the new unbiased Pohar-Perme estimator and magnitude of the bias with the classical methods. Int J Cancer. 2013;132:2359–69. 22961565 10.1002/ijc.27830

[25] Perme MP, Pavlic K. Nonparametric relative survival analysis with the R package relsurv. J Stat Softw. 2018;87:1–27.

[26] Sinclair DR, Maharani A, Chandola T et al. Frailty among older adults and its distribution in England. J Frailty Aging. 2022;11:163–8. 35441193 10.14283/jfa.2021.55

[27] Crow RS, Lohman MC, Titus AJ et al. Association of Obesity and Frailty in older adults: NHANES 1999-2004. J Nutr Health Aging. 2019;23:138–44. 30697622 10.1007/s12603-018-1138-x PMC6371801

[28] Di Girolamo C, Walters S, Benitez Majano S et al. Characteristics of patients with missing information on stage: A population-based study of patients diagnosed with colon, lung or breast cancer in England in 2013. BMC Cancer. 2018;18:492. 29716543 10.1186/s12885-018-4417-3 PMC5930770

[29] Pilleron S, Withrow DR, Nicholson BD, Morris EJA . Age-related differences in colon and rectal cancer survival by stage, histology, and tumour site: An analysis of United States SEER-18 data. Cancer Epidemiol. 2023;84:102363. 10.1016/j.canep.2023.102363 37060832

[30] Pilleron S, Maringe C, Charvat H et al. The impact of timely cancer diagnosis on age disparities in colon cancer survival. J Geriatr Oncol. 2021;12:1044–51. 33863698 10.1016/j.jgo.2021.04.003

