# Supplementary material for: Age-related differences in staging, treatment and net survival in relation to frailty in adults with colon cancer in England: an analysis of the COloRECTal cancer data repository (CORECT-R) resource
Source: Age Ageing. 2025 Feb 19;54(2):afaf025. doi: 10.1093/ageing/afaf025 (PMC11836420; doi:10.1093/ageing/afaf025)
Supplement: aa-24-0997-File003_afaf025 [file aa-24-0997-file003_afaf025.docx]

**Age-related differences in staging, treatment, and net survival in relation to frailty in adults with colon cancer in England: an analysis of the COloRECTal cancer data Repository (CORECT-R) resource**

**Content**

| List of items used in the SCARF index | Page 2 |
| --- | --- |
| Supplemental Table – 1-year net survival and 1-year net survival conditional on surviving the 1^st^ year of diagnosis by age group and SCARF frailty levels in adults diagnosed with colon cancer in England, 2014-2019 | Page 3 |
| Supplemental Figure 1 – One-year net survival conditioning on surviving the first year of diagnosis by age group and SCARF frailty level | Page 4 |
| R codes | Page 5 |

**List of items used in the SCARF index.**

*Source: Jauhari Y, Gannon MR, Dodwell D, Horgan K, Clements K, Medina J, et al. Construction of the secondary care administrative records frailty (SCARF) index and validation on older women with operable invasive breast cancer in England and Wales: a cohort study. BMJ Open. 2020 May 5;10(5):e035395.*

**Functional impairment**

Activity limitations

Neurodegenerative disorders

Requirement for care

Social vulnerability

Hearing impairment

Visual impairment

**Geriatric syndrome**

Falls

Skin ulcer

Incontinence

**Comorbidities**

Anaemia

Arthritis

Cardiac arrhythmia

Cerebrovascular disease

Chronic kidney disease

Diabetes

Diabetic complications

Heart failure

Heart valve disease

Hypertension

Hypotension

Ischaemic heart disease

Foot problems

Fragility fracture

Osteoporosis

Peptic ulcer

Peripheral vascular disease

Respiratory disease

Thyroid disease

Urinary system diseases

**Nutritional Problem**

**Cognitive and mental health problems**

**Supplemental Table –** 1-year net survival and 1-year net survival conditional on surviving the 1^st^ year of diagnosis by age group and SCARF frailty levels in adults diagnosed with colon cancer in England, 2014-2019

| **Age groups** | **50-64** | **65-74** | **75-84** | **85-99** |
| --- | --- | --- | --- | --- |
| **1-year net survival from diagnosis** | | | | |
| Fit | 87.3 (86.8-87.8) | 85.7 (85.2-86.2) | 77.7 (77.0-78.4) | 59.0 (57.6-60.4) |
| Mildly frail | 80.1 (79.0-81.2) | 79.2 (78.3-80.1) | 75.4 (74.5-76.4) | 63.6 (61.8-65.4) |
| Moderately frail | 76.4 (74.5-78.2) | 73.5 (72.2-74.9) | 70.6 (69.4-71.7) | 54.9 (53.1-56.8) |
| Severely frail | 69.8 (67.1-72.7) | 65.9 (64.3-67.5) | 57.1 (55.9-58.2) | 40.9 (39.5-42.2) |
| **1-year net survival conditional on surviving the 1^st^ year of diagnosis** | | | | |
| All | 90.0 (89.6-90.4) | 90.8 (90.5-91.2) | 89.1 (88.6-89.6) | 81.2 (80.1-82.2) |
| Fit | 91.2 (90.7-91.7) | 92.8 (92.3-93.2) | 92.1 (91.4-92.8) | 85.3 (83.5-87.0) |
| Mildly frail | 87.6 (86.5-88.6) | 89.7 (88.9-90.6) | 90.4 (89.5-91.3) | 86.5 (84.4-88.6) |
| Moderately frail | 87.4 (85.7-89.1) | 88.4 (87.1-89.6) | 88.2 (87.0-89.3) | 79.2 (76.7-81.8) |
| Severely frail | 82.2 (79.5-85.1) | 82.1 (80.5-83.8) | 79.9 (78.5-81.3) | 71.6 (69.3-74.0) |

**Supplemental Figure**


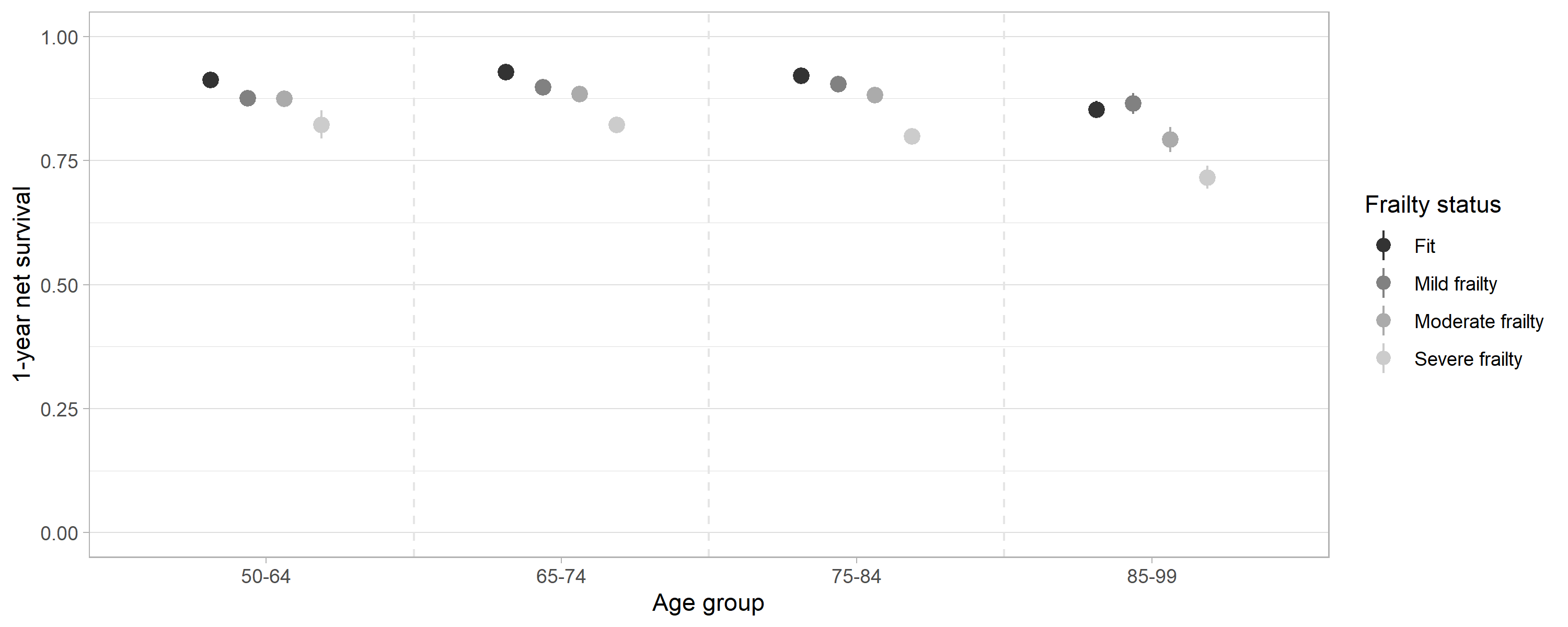


**Supplemental Figure 1** – One-year net survival conditioning on surviving the first year of diagnosis by age group and SCARF frailty level

**R codes**

**R Code 1** - **Preparation of data for analysis**

## Input: Data cut from CORECT-R team - 106 298 patients

## Output: Dataset for analysis + info for flowchart

##UPDATE: New data cut on November 2022 - 130 377 patients

# Libraries ----------------------------------------------------------------

library(haven);library(Hmisc)

library(readstata13)

# Original datasets ----------------------------------------------------------------

path <- "Your path"

data <- read.dta13(paste0(path,"Extract_file.dta"),nonint.factors = TRUE)

# colnames(data)

# describe(data)

# Set the working folder

setwd("My folder »)

# First look --------------------------------------------------------------

# range(data$age) # 50-99

# range(data$yod) # 2014-2018 - new cut up to 2019

# range(data$vitstatdt,na.rm=T) # "2014-01-02" "202-03-01" - new cut: "2014-01-01" "2022-09-21"

# sum(duplicated(data$tumourid)) # no duplicates

# sum(duplicated(data$patientid)) # no duplicates

# Analysis datasets ----------------------------------------------------------------

# d <- data[,c("patientid","yod","imd2015", "age", "inc_dt", "ethnicityname", "sex"

# , "vitalstatus", "vitstatdt","usestage","site4","proctype","basisofdiagnosis"

# ,"route_f","c3_total","c3_cat","scarf_score","scarf_cat")]

d <- data[,c("patientid","yod","imd2015", "age", "inc_dt", "sex"

, "vitalstatus", "vitstatdt","usestage","proctype","scarf_score","scarf_cat")]

d$sex <- ifelse(d$sex=="M","1","2")

n <- dim(d)[1] # 130377

describe(d)

# Vital status

# X lost to follow up

# D dead

table(d$vitalstatus)

d$status <- d$vitalstatus

d$status <- ifelse(d$status%in%c("D3","D4","D5"),"D",d$status)

# d$status <- ifelse(d$status%in%c("X","X2"),NA,d$status)

d$status <- ifelse(d$status%in%c("X2","X4","X5"),"A",d$status) # X considered as alive and censored at date of last FU

# table(d$status,useNA = "always")

table(d$status,d$vitalstatus,useNA = "always")

#lostFU <- sum(d$vitalstatus%in%c("X","X2"))

# Exclude if vital status missing

d <- d[d$status%in%c("D","A"),]

n <- c(n,dim(d)[1])

# Exclude if vital status date missing

d <- d[!is.na(d$vitstatdt),]

n <- c(n,dim(d)[1])

# Survival time - set end of FU on Dec 31,2021 because lifetable go up to 2021

endFU <- as.Date("2021-12-31","%Y-%m-%d")

d$endFU <- ifelse(d$vitstatdt<=endFU,d$vitstatdt,endFU)

d$endFU <- as.Date(d$endFU,origin = "1970-01-01")

# Change status accordingly

d$status <- ifelse(d$status=="D" & d$vitstatdt>endFU,"A",d$status)

d$surv <- d$endFU - d$inc_dt

# Survival time in years

d$time_n <- d$surv/365.241

head(d)

n <- c(n,dim(d)[1])

# # Exclude if dco

# d <- d[d$route_f!="DCO",]

# n <- c(n,dim(d)[1])

# Recode NAs for frailty with 0

d$frailty <- as.factor(d$scarf_cat)

# Ethnicity - 90.7 per cent White

# Re-coding of deprivation

table(d$imd2015,useNA = "always")

d[d$imd2015=="1 - least deprived",]$imd2015 <- "1"

d[d$imd2015=="5 - most deprived",]$imd2015 <- 5

d$imd2015 <- as.numeric(d$imd2015)

d$imd2015 <- as.factor(d$imd2015)

## Merge with life table - https://www.cancerdata.nhs.uk/survival/lifetables

load("./lt.RData")

head(d)

head(lt.nat)

colnames(d)[2:3] <- c("year","dep")

lt.nat$age <- lt.nat$age/365.25

range(lt.nat$age)

lt.nat$sex <- ifelse(lt.nat$sex=="Female",2,1)

Mybase <- merge(d, lt.nat,by = c("age","sex","dep","year"),all=T)

Mybase <- Mybase[!is.na(Mybase$patientid),]

# Create age categories

Mybase$agegp <- cut(Mybase$age,c(40,64,74,84,100))

# Create a variable for major resection for patients with stage I-III colon cancer only

Mybase$surgery <- ifelse(Mybase$proctype==1,"1","0")

Mybase$surgery <- ifelse(Mybase$usestage%in%c("IV","U"),NA,Mybase$surgery) # only in patients with stage I-III

## Sample selection

flow <- paste0("Colon cancer cases diagnosed in 2014-2019 (n=",n[1],")")

flow <- paste0(flow,"\n", "with vital status (n=",n[2],")")

flow <- paste0(flow,"\n", "with vital status date (n=",n[3],")")

flow <- paste0(flow,"\n", "with survival (n=",n[4],")")

flow <- paste0(flow,"\n", "% cases excluded = ",round(100-((n[4]*100)/n[1]),1),"%")

cat(flow)

## Save dataset for future use

save(flow,Mybase,lt.nat,file="./Mybase.RData")

# END ---------------------------------------------------------------------

**R Code 2 – Estimating 1-year Net survival & 1-year NS conditional on surviving 1 year after diagnosis**

# Packages ----------------------------------------------------------------

library(survival); library(mexhaz); library(splines); library("relsurv");library(lubridate)

# Load data + useful code ---------------------------------------------------------------

# Set the working folder

setwd("My folder")

# Upload working dataset prepared by "1- PrepaDataset_April2024.R" - CAN BE SKIPPED IF DATA ALREADY UPLOADED

load("./Mybase.RData")

# Load code written by H. Charvat to help to use relsurv package

source("My Path/Imports/PetitProg.R")

# Net survival estimation -------------------------------------------------

# Rename variables of lifetable (ie. lt.nat)

lt <- lt.nat[,-c(5)]

colnames(lt)[5] <- c("prob")

# Convert the lifetable into a ratetable requested by relsurv package

t <- ConvertMultLT(lt,strat = "dep",year = "year",sex="sex",int.length = 1)

# Some recoding

Mybase$sex <- as.numeric(Mybase$sex)

Mybase$agegp <- cut(Mybase$age,c(40,64,74,84,100))

# Censor at 1 year as interested in 1-year survival

Mybase$surv2 <- ifelse(Mybase$surv>365.241,365.241,Mybase$surv)

Mybase$status2 <- ifelse(Mybase$surv>365.241 & Mybase$status=="D","A",Mybase$status)

# Recode frailty and agegp categories to make Fig 4 easier to create

Mybase$frailty.n <- NA

Mybase[Mybase$frailty=="Fit",]$frailty.n <- "a"

Mybase[Mybase$frailty=="Mild frailty",]$frailty.n <- "d"

Mybase[Mybase$frailty=="Moderate frailty",]$frailty.n <- "c"

Mybase[Mybase$frailty=="Severe frailty",]$frailty.n <- "b"

Mybase$agegp.n <- NA

Mybase[Mybase$agegp=="(40,64]",]$agegp.n <- "age1"

Mybase[Mybase$agegp=="(64,74]",]$agegp.n <- "age4"

Mybase[Mybase$agegp=="(74,84]",]$agegp.n <- "age3"

Mybase[Mybase$agegp=="(84,100]",]$agegp.n <- "age2"

# 1- year Net Survival by age group

# Model to get net survival estimate

mod.all <- rs.surv(Surv(surv2,event=status2=="D") ~ agegp.n, rmap=list(age=age*365.241),add.times=c(0.5,1)*365.241

,method = "pohar-perme", ratetable = t,data=Mybase)

# Object to store estimates

res.all <- summary(mod.all,times =c(1)*365.241 )

# 1- year Net Survival by age group and frailty

# Model to get net survival estimate

mod <- rs.surv(Surv(surv2,event=status2=="D") ~ agegp.n*frailty.n, rmap=list(age=age*365.241),add.times=c(0.5,1)*365.241

,method = "pohar-perme", ratetable = t,data=Mybase)

# Object to store estimates

res <- summary(mod,times =c(1)*365.241 )

# 1-year NS conditioning on surviving 1-year of diagnosis

# Keep only those who survived 1 y

dt.cond <- Mybase[Mybase$surv>=365.241,]

dim(dt.cond) # 90078

range(dt.cond$surv) # 366 - 2921

# Subtract 365.241 days to total survival time

dt.cond$condsurv <- dt.cond$surv - 365.241

range(dt.cond$condsurv) # 0.759 - 2555.759

# Censor survival time at 1 year and re-code vital status accordingly

dt.cond$condsurv2 <- ifelse(dt.cond$condsurv>365.241,365.241,dt.cond$condsurv)

dt.cond$status2 <- ifelse(dt.cond$condsurv>365.241 & dt.cond$status=="D","A",dt.cond$status)

# Model to estimate conditional survival by age group

mod.cond.all <- rs.surv(Surv(condsurv2,event=status2=="D") ~ agegp.n, rmap=list(age=age*365.241),add.times=365.241

,method = "pohar-perme", ratetable = t,data=dt.cond)

# Object to store survival estimates

res.cond.all <- summary(mod.cond.all, times=365.241)

# Model to estimate conditional survival by age group and frailty

mod.cond <- rs.surv(Surv(condsurv2,event=status2=="D") ~ agegp.n*frailty.n, rmap=list(age=age*365.241),add.times=365.241

,method = "pohar-perme", ratetable = t,data=dt.cond)

# Object to store survival estimates

res.cond <- summary(mod.cond, times=365.241)

save(flow,Mybase,lt.nat, res, res.all, res.cond.all, res.cond,file="./Mybase.RData")

# END ---------------------------------------------------------------------

**R Code 3 - Creating Tables 1 & 2**

# Packages

library(tableone)

# Set the working folder

setwd("My folder")

# Upload working dataset prepared by "1- PrepaDataset_Nov2022.R" - CAN BE SKIPPED IF DATA ALREADY UPLOADED

load("./Mybase.RData")

# Creation of some variables

Mybase$status2 <- ifelse(Mybase$surv>365.241 & Mybase$status=="D","A",Mybase$status) # number of deaths

Mybase$age75 <- cut(Mybase$age,c(40,74,100))

table(Mybase$age75)

## Convert categorical variables into factors

varsToFactor <- c("sex","dep","frailty","surgery")

Mybase[varsToFactor] <- lapply(Mybase[varsToFactor], factor)

## Create a variable list

vars <- c("status2","age","age75","sex","dep",

"usestage","frailty","surgery")

## Create Table for overall population

tableOne_all <- CreateTableOne(vars = vars,test=F,data = Mybase)

#

print(tableOne_all, nonnormal = c("age"),quote=T,noSpaces = T)

## Create Table 1 stratified by agegp

tableOne <- CreateTableOne(vars = vars, strata = c("agegp"), test=F,data = Mybase)

print(tableOne, nonnormal = c("age"),quote=T,noSpaces = T)

## Complementary analysis - % (minor& major) surgery by frailty status in unstaged cancers

# proctype =1 --> major / =2 --> minor / =6 --> no surgery

Mybase$unkstagebutsurg <- ifelse(Mybase$usestage=="U" & Mybase$proctype==1,"1","0")

Mybase$unkstagebutsurg <- ifelse(Mybase$usestage!="U",NA,Mybase$unkstagebutsurg)

# table(Mybase$unkstagebutsurg)

# table(Mybase$unkstagebutsurg,Mybase$frailty)

#tab <- CreateTableOne(vars = "unkstagebutsurg", strata = c("agegp"), test=F,data = Mybase)

#print(tab)

#tab2 <- CreateTableOne(vars = "unkstagebutsurg", strata = c("agegp","frailty"), test=F,data = Mybase)

#print(tab2)

#Mybase$unkstagenosurg <- ifelse(Mybase$usestage=="U" & Mybase$proctype==6,"1","0")

#Mybase$unkstagenosurg <- ifelse(Mybase$usestage!="U",NA,Mybase$unkstagenosurg)

#tab3 <- CreateTableOne(vars = "unkstagenosurg", strata = c("agegp","frailty"), test=F,data = Mybase)

#print(tab3)

# Table 2

Mybase$unkstagesurg1_2 <- ifelse(Mybase$usestage=="U" & Mybase$proctype%in%c(1,2),"1","0")

Mybase$unkstagesurg1_2 <- ifelse(Mybase$usestage!="U",NA,Mybase$unkstagesurg1_2)

tab4 <- CreateTableOne(vars = "unkstagesurg1_2", strata = c("agegp","frailty"), test=F,data = Mybase)

print(tab4)

save(flow,Mybase,lt.nat, res, res.all, res.cond.all, res.cond,file="./Mybase.RData")

# END ---------------------------------------------------------------------

**R code 4 - Creating Figures 1-4 in the main paper + Figure in supplemental material**

# Packages

library(tableone) ; library(ggplot2) ; library("ggpubr")

# Set the working folder

setwd("My folder")

# Upload working dataset prepared by "1- PrepaDataset_Nov2022.R" - CAN BE SKIPPED IF DATA ALREADY UPLOADED

load("My path »/Mybase.RData")

# Fig 1 - Stacked plot - frailty by age group -------------------------------------

tableOne <- CreateTableOne(vars = "frailty", strata = c("agegp"), test=F,data = Mybase)

tableOne

dt <- expand.grid(frailty=c("Fit","Mild frailty","Moderate frailty","Severe frailty")

,agegp=c("50-64","65-74","75-84","85-99")

,freq=NA,percent=NA)

dt[1:4,3] <- tableOne$CatTable$`(40,64]`$frailty$freq

dt[5:8,3] <- tableOne$CatTable$`(64,74]`$frailty$freq

dt[9:12,3] <- tableOne$CatTable$`(74,84]`$frailty$freq

dt[13:16,3] <-tableOne$CatTable$`(84,100]`$frailty$freq

dt[1:4,4] <- tableOne$CatTable$`(40,64]`$frailty$percent

dt[5:8,4] <- tableOne$CatTable$`(64,74]`$frailty$percent

dt[9:12,4] <- tableOne$CatTable$`(74,84]`$frailty$percent

dt[13:16,4] <-tableOne$CatTable$`(84,100]`$frailty$percent

dt$frailty <- as.character(dt$frailty)

dt$agegp <- as.character(dt$agegp)

fig1 <- ggplot(dt,aes(fill=frailty,x=agegp,y=freq))+

geom_bar(position="fill",stat = "identity")+

xlab("Age group")+

ylab("Percentages")+

scale_fill_grey()+

theme_light()+

labs(fill="Frailty status")+

ggtitle("A - Distribution of SCARF categories by age group")+

geom_text(aes(y=dt$freq,label=sprintf("%0.1f",round(dt$percent,1)),color=frailty),

position=position_fill(vjust = 0.5))+

scale_colour_manual(values=c("White",rep("Black",3))) +

guides(color=FALSE)

fig1

# Fig 2 - % stage by frailty ------------------------------------------------------

tableOne <- CreateTableOne(vars = "usestage", strata = c("frailty","agegp"), test=F,data = Mybase)

tableOne

dt.stage <- expand.grid(stage=c("I","II","III","IV","Unknown")

,frailty=c("Fit","Mild","Moderate","Severe")

,agegp=c("50-64","65-74","75-84","85-99")

,perc=NA)

# sort dt.stage manually

for (i in 0:15){

dt.stage[(i*5) + (1:5),4] <- round(tableOne[["CatTable"]][[i+1]][["usestage"]][["percent"]],1)

}

fig2 <- ggplot(dt.stage,aes(fill=stage,x=frailty,y=perc))+

geom_bar(position="fill",stat = "identity")+

xlab("SCARF categories")+

ylab("Percentages")+

scale_fill_grey()+

theme_light()+

labs(fill="Stage at diagnosis")+

ggtitle("B - Distribution of stage by SCARF category and age group")+

geom_text(aes(y=dt.stage$perc,label=sprintf("%0.1f",round(dt.stage$perc,1)),color=stage),

position=position_fill(vjust = 0.5))+

facet_grid(~ agegp)+

scale_colour_manual(values=c("White",rep("Black",4)))+

guides(color=FALSE)

fig2

# Fig 3 - Major Surgery by age and frailty ----------------------------------------

tableOne <- CreateTableOne(vars = "surgery", strata = c("agegp","frailty"), test=F, data = Mybase[Mybase$usestage%in%c("I","II","III"),])

print(tableOne)

dt.surg.2 <- expand.grid(agegp=c("50-64","65-74","75-84","85-99"),frailty=c("Fit","Mild frailty","Moderate frailty","Severe frailty")

,perc=NA)

for (i in 1:16){

dt.surg.2[i,3] <- round(tableOne[["CatTable"]][[i]][["surgery"]][["percent"]][2],1)

}

fig3 <- ggplot(dt.surg.2,aes(x=agegp,y=perc,fill=frailty))+

geom_col(position=position_dodge())+

ylim(c(0,100))+

xlab("Age group")+

ylab("Percentages")+

scale_fill_grey()+

theme_light()+

labs(fill="Frailty status")+

ggtitle("C - Distribution of receipt of major surgery in patients with stage I-III colon cancer⁠\n⁠by SCARF category and age group")+

geom_text(aes(label=sprintf("%0.1f",round(perc,1)),color=frailty),

position=position_dodge(width=0.9),vjust=1.6)+

scale_colour_manual(values=c("White",rep("Black",3)))+

guides(color=FALSE)

fig3

# Fig 4 - 1-year NS by age group and frailty --------------------------------------

dt.ns <- expand.grid(frailty=c("Fit","Mild frailty","Moderate frailty","Severe frailty")

,agegp=c("50-64","65-74","75-84","85-99")

,surv=0,low=0,high=0)

dt.ns[,3] <- res[7]

dt.ns[,4] <- res[9]

dt.ns[,5] <- res[10]

fig4 <- ggplot(dt.ns,aes(x=agegp,y=surv,colour=frailty))+

ylim(0,1)+

geom_pointrange(aes(ymin=low,ymax=high),

fatten = 5,

position = position_dodge(width=0.5))+

xlab("Age group")+

ylab("1-year net survival")+

scale_colour_grey()+

theme_light()+

labs(color="Frailty status")+

ggtitle("D - 1-year net survival by SCARF category and age group")+

theme(panel.grid.major.x = element_blank())+

geom_vline(xintercept = c(1.5,2.5,3.5),colour = "grey90",

linetype="dashed")

fig4

save(flow,Mybase,lt.nat,fig1, fig2, fig3, fig4, file="./Mybase.RData")

# 1-year NS conditioning on surviving 1 year by age and frailty -----------

dt.cond.ns <- expand.grid(frailty=c("Fit","Mild frailty","Moderate frailty","Severe frailty")

,agegp=c("50-64","65-74","75-84","85-99")

,surv=NA,low=NA,high=NA)

dt.cond.ns[,3] <- res.cond[7]

dt.cond.ns[,4] <- res.cond[9]

dt.cond.ns[,5] <- res.cond[10]

fig5 <- ggplot(dt.cond.ns,aes(x=agegp,y=surv,colour=frailty))+

ylim(0,1)+

geom_pointrange(aes(ymin=low,ymax=high),

fatten = 5,

position = position_dodge(width=0.5))+

xlab("Age group")+

ylab("1-year net survival")+

scale_colour_grey() +

theme_light()+

labs(color="Frailty status")+

theme(panel.grid.major.x = element_blank())+

geom_vline(xintercept = c(1.5,2.5,3.5),colour = "grey90",

linetype="dashed")+

fig5

# Arrange plots main paper -----------------------------------------------------------

Fig.all <- ggarrange(fig1,fig2,fig3,fig4,ncol=2,nrow=2)

Fig.all

ggsave("Fig.png",Fig.all, width=20,height=8, dpi=300)

# Save supplemental Figure ------------------------------------------------

ggsave("SupplFig.png",fig5, width=20,height=8, dpi=300)

save(flow,Mybase,lt.nat, res, res.all, res.cond.all, res.cond, fig1, fig2, fig3, fig4, fig5, file="./Mybase.RData")

##############################################
